# Synthesis of a New Multifunctional Ionite Based on DBA-EChH-PEI and Its Efficiency in the Sorption of Copper and Silver Ions

**DOI:** 10.3390/polym17243287

**Published:** 2025-12-11

**Authors:** Yevgeniy Melnikov, Xeniya Morozova, Ruslan Kondaurov

**Affiliations:** Biochemical Engineering Department, International Engineering and Technological University, Al-Farabi Ave. 93a, Almaty 050060, Kazakhstan; sebas273@mail.ru (Y.M.); ksusamorozova05@mail.ru (X.M.)

**Keywords:** sorption, ion-exchange, silver ions, copper ions, ionometry, sorption capacity

## Abstract

A comparative sorption dependence was carried out between the Dowex HCR-S/S″ industrial ion-exchange sorbent and the synthesized ion-exchange sorbent based on dibenzylamine, epichlorohydrin and polyethylenimine in relation to copper and silver ions. The sorption of copper and silver was studied by ionometry and the dependences of the sorption of copper and silver ions in the static mode were established depending on the concentration of metal ions and the duration of ionite contact with solutions of copper and silver nitrates. It was found that the maximum sorption capacity of the synthesized ion exchanger is 672.4 mg/g for copper ions and 721.0 mg/g for silver ions, and 626.3 mg/g and 679.7 mg/g for industrial Dowex HCR-S/S″ ionite, respectively. It is shown that the sorption of copper and silver is described by various kinetic models: for copper, the best correspondence is demonstrated by a pseudo second order kinetic model, whereas for silver, the Elovich kinetic model the different nature of the interaction of ions with active centers. It has been revealed that the synthesized ion exchanger is superior to an industrial sorbent in terms of sorption rate and degree of extraction of valuable metals, especially in concentrated solutions, which indicates the prospects of its use in the processes of selective extraction of copper and silver.

## 1. Introduction

Copper and silver are essential industrial metals that play a critical role in modern technologies, including electrical engineering, microelectronics, catalysis, energy systems, antimicrobial coatings, and chemical manufacturing. Their unique physicochemical properties—high electrical and thermal conductivity, catalytic activity, and biological reactivity—determine the growing global demand for efficient extraction, recovery, and purification processes. As the consumption of these metals increases steadily, the need for sustainable management of mineral, technological, and secondary resources becomes ever more urgent [1,2,3].

Kazakhstan is among the world’s leading countries in terms of copper reserves and possesses a diverse and extensive raw material base. The Zhezkazgan basin, which has large porphyry-type deposits such as Aktogay and Koksay, and the Bozshakol open-pit mine represent the major operational and prospective ore regions. Their cumulative resources demonstrate significant copper reserves accompanied by valuable by-products such as gold, silver, molybdenum, and rare metals. Silver in Kazakhstan typically does not occur as an independent ore mineral; instead, it is associated with sulfide and polymetallic ores, including chalcopyrite, galena, arsenide phases, and sulfosalts. In some sectors of the Zhezkazgan ore field, locally elevated silver contents reaching tens of grams per ton are recorded, which increases the importance of comprehensive extraction schemes [4,5,6,7,8].

Despite the abundance of deposits, the mining industry faces a gradual decrease in the quality of primary mineral raw materials. Average copper contents often do not exceed 0.3–0.4%, and richer zones exceeding 1% Cu in sedimentary-associated deposits are becoming increasingly rare. A similar trend characterizes the silver industry: although global silver production continues to grow by 1.5–2 thousand tons annually, existing reserves are finite. Forecasts based on current consumption rates suggest that economically viable silver resources may be depleted within the next two decades. Thus, the depletion of high-grade deposits, increasing depth of ore bodies, and the need to process complex mineralogical types raise the cost and difficulty of traditional metallurgy [9,10,11,12,13,14,15,16].

At the same time, the processing of low-grade polymetallic ores intensifies environmental pressure. In Kazakhstan, where over half of metal-containing raw materials are extracted by open-pit mining, hydrometallurgical processes contribute to soil degradation, vegetation loss, contamination of natural waters with heavy metals and processing reagents, and the migration of toxic components in the biosphere. These factors underscore the necessity of developing environmentally safe, resource-efficient, integrated technologies aimed at the sustainable extraction and recycling of valuable metals [17,18,19].

Against this background, secondary raw materials—including technological process solutions, tailings, and industrial wastewater—are becoming a strategically important source of copper, silver, and other critical metals. The recovery of Cu(II), Ag(I), and rare-earth ions from aqueous streams is in full alignment with global trends toward circular economy, reduction in primary resource dependence, and minimization of environmental impact. Industrial solutions generated by mining, metallurgical, and electronic-waste processing plants often contain significant quantities of recoverable metals, making them attractive objects for selective purification and extraction [20,21,22,23,24,25,26].

Among the numerous physicochemical methods proposed for the removal and recovery of metal ions from aqueous media, sorption is one of the most effective and technologically flexible approaches. Modern sorption systems include activated carbons, inorganic oxides, hybrid composites, and a broad range of functionalized polymeric materials. Polymer sorbents, in particular, have gained substantial attention due to their high structural tunability, chemical and mechanical resilience, reusability, and ability to incorporate targeted functional groups with high affinity for specific metal ions. Nitrogen-containing ligands such as primary, secondary, and tertiary amines significantly enhance chelation efficiency toward Ag(I) and Cu(II), making such polymers especially suitable for selective metal recovery [27,28,29,30,31].

A promising strategy for designing advanced ion-exchange materials is the synergistic combination of dibenzylamine (DBA), epichlorohydrin (EChH), and polyethyleneimine (PEI). Polyethyleneimine provides a high density of amino groups responsible for metal coordination; epichlorohydrin serves as a crosslinking agent, imparting chemical stability, rigidity, and resistance to hydrolysis; while DBA introduces steric and hydrophobic elements that can positively influence metal binding kinetics and selectivity. These structural features create conditions for engineering multifunctional hybrid polymer matrices with enhanced sorption capacity, improved kinetic behavior, and increased resistance to aggressive environments. Such materials have potential applications not only in the extraction of non-ferrous and noble metals but also in catalysis, chromatographic separation, environmental remediation, and sensor technologies.

Considering these factors, the present study addresses the urgent need for efficient sorbents capable of recovering copper(II) and silver(I) ions from aqueous systems of varying concentrations. A new ion-exchange polymer based on DBA–EChH–PEI was synthesized and systematically investigated. Its sorption kinetics and extraction efficiency toward Cu^2+^ and Ag^+^ ions were examined under controlled laboratory conditions and directly compared with the performance of the commercial ionite Dowex HCR-S/S″. Particular attention was paid to the influence of contact time and initial ion concentration, enabling a comprehensive assessment of kinetic parameters and diffusion characteristics.

The scientific novelty of the work lies in the synthesis of a previously unreported DBA–EChH–PEI polymer ion-exchange material and its comparative evaluation with an industrial analog. The determination of concentration-dependent kinetic constants and extraction efficiency provides a foundation for quantitatively predicting sorption behavior and optimizing operational conditions for real technological and wastewater treatment processes. This research thus contributes to the development of effective polymer sorbents for sustainable metal recovery and supports broader efforts toward environmentally responsible resource management.

## 2. Materials and Methods

### 2.1. Materials

Dowex HCR-S/S″ produced by “The Dow Chemical Company” (Midland, MI, USA). It is an industrial highly acidic gel-type cation exchange resin based on a sulfonated divinylbenzene styrene copolymer.


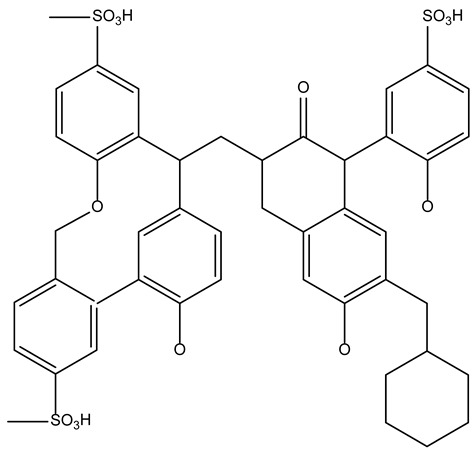


DBA–EChH–PEI is a synthesized anion exchange resin based on dibenzylamine, epichlorohydrin and polyethylenimine.


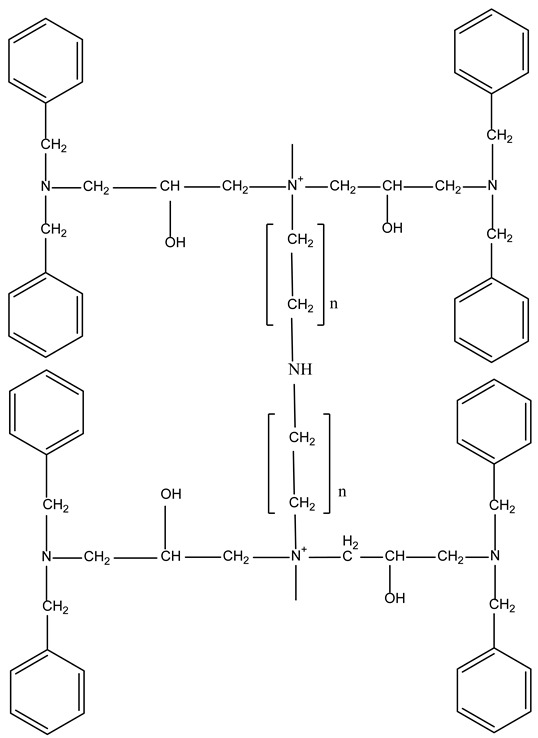


Silver nitrate (I) produced by “Sigma-Aldrich” (Burlington, MA, USA) with a composition of main product ≥ 99% was used without pre-treatment.

Copper Nitrate (II) produced by “Sigma-Aldrich” (Burlington, MA, USA) with a composition of main product ≥ 99% was used without pre-treatment.

### 2.2. Synthesis of Ion Exchange Resin

Epoxyamine was synthesized from dibenzylamine (DBA) and epichlorohydrin (EChH), which was condensed with polyethylenimine (PEI) to produce a multifunctional DBA–EChH–PEI anionite. First, a glycidyl derivative of an amine (epoxyamine) was synthesized from DBA and ECG in the presence of caustic soda at a temperature of 50–60 °C for 6 h. Then it was polycondensed with PEI in a dimethylformamide solution at different mass ratios, at a temperature of 60–65 °C and a duration of 5–6 h, after which the reaction mass was cured at 100 °C for 16–24 h. Soluble polyamine with a large number of amino groups, polyethylenimine, was used to synthesize anionites based on them. Amination of dibenzylamine was carried out in a dimethylformamide medium, the use of which prevented local overheating due to the exothermic effect of the opening of epoxy groups. When diglycidyldibenzylamine interacts with polyamines, sequential nucleophilic attachment of amino groups to epoxy groups occurs. As the degree of transformation increases, the rate of the polycondensation reaction decreases significantly due to a decrease in the mobility and accessibility of functional groups during the formation of spatial structure products [32]. As a result, a macro-lattice multifunctional anionite DBA-EHG-PEI was obtained. The effect of the temperature and duration of the polycondensation reaction of diglycidyl derivatives and polyamines on the properties of anionites was studied: from 40 to 80 °C, and the synthesis time from 2 to 8 h. The optimal reaction time and duration were 60–65 °C and 5–6 h.

### 2.3. Physicochemical Methods of Analysis

Static exchange capacity (SEC) was determined as follows: a sample of anionite in OH form in the amount of 1 g per dry substance, weighted to an accuracy of 0.0002 g, was poured into 100 mL of 0.1 n HCl solution in a flat-bottomed flask with a capacity of 250 mL and tightly closed with a stopper. After equilibrium was established (24 h), 25 mL of the filtrate was titrated with 0.1 n NaOH solution in the presence of three drops of the mixed indicator until the blue color turned green.

SEC = (100 − 4 V)/10 g

where V is the volume of exactly 0.1 n sodium hydroxide solution used for titration (mL); g is the weight of ionite in terms of dry matter, g.

To determine the volume occupied by a unit mass of dry ionite after swelling in an aqueous medium, a weight of about 10 g was placed in a cylinder and 70 mL of water was poured. The cylinder was tightly closed with a stopper, shaken until the lower ionite layers were completely wetted and left in a horizontal position for 12 h. Then, the cylinder was returned to the vertical position, and up to 100 mL of water was added and compacted to a constant volume by tapping the bottom of the cylinder on a wooden surface. After compaction, the volume occupied by the ionite was measured. The specific volume of ionite in the swollen state (Vud., mL/g) was calculated by the formula:

V_sp_ = V/g,

where V is the volume of the swollen ionite, mL; g is the suspension of dry ionite, g.

The chemical stability of ionite to solutions of acids and alkalis was determined as follows: two ionite weights of 0.1 g each, in terms of dry matter, were taken and placed in round-bottomed flasks with a return refrigerator with a capacity of 250 mL. One sample was filled with 100 mL of 5 n sulfuric acid solution, the other with 100 mL of 5 n sodium hydroxide solution. The contents of the flasks were kept in a boiling water bath for 30 min. The mixture was then cooled in air to room temperature and the ionite was separated by filtration. If necessary, the anionite was converted to the hydroxyl form. The ionites were washed with distilled water and their static exchange capacity was determined using 0.1 n hydrochloric acid solution.

CS = SEC/SEC_0_ × 100,

where SEC_0_ and SEC are the static exchange capacity of ionites before and after treatment with acid or alkali.

The chemical stability of ionite in relation to solutions of oxidizing agents was determined by the following procedure: a sample of ionite (1 g) was taken, 100 mL of 10% hydrogen peroxide solution was poured, and kept at room temperature for 48 h with periodic stirring. Ionite was separated by filtration, converted to hydroxyl form, washed with distilled water and its static exchange capacity was determined by 0.1 n hydrochloric acid solution.

To determine the thermal stability of ionites, a sample of ionite (2–3 g) was placed in a round-bottomed flask with a 250 mL reflux refrigerator, filled with 100 mL of water and kept in a boiling water bath for 50 h. After cooling to room temperature, the ionite was separated by filtration and washed with distilled water, and the static exchange capacity was determined using 0.1 n hydrochloric acid solution.

TS = SEC/SEC_0_ × 100

where SEC_0_ and SEC are the static exchange capacity of ionites before and after boiling.

The sorption of silver and copper ions by sorbents was studied under static conditions at a sorbent:solution ratio of 1:400 at room temperature of 20 ± 2 °C, varying the concentration of silver ions in AgNO_3_ solutions from 0.256 to 2.050 g/L and Cu(NO_3_)_2_ from 0.156 to 1.980 g/L. The duration of contact of sorbents with solutions ranged from 1.0 h to 7 days. The sorption capacity was calculated from the difference between the initial and equilibrium concentrations of solutions, which were determined by ionometry on an I-160MI device. The error of the concentration determination method (3–5%) contributes to the dispersion of data by isotherms. For a pair of ionites, where the difference in capacitance is about 7–8%, the error of the method makes this difference bordering on significant. The sorption properties of these two ionites are statistically indistinguishable within the framework of the methodology used.

## 3. Results and Discussion

Table 1 shows some of the studied physico-chemical characteristics of the synthesized anionite. The synthesized DBA-EChH-PEI ionite demonstrated a 7–10% higher sorption capacity and ~12% better chemical stability to acids and alkalis compared to Dowex HCR-S/S″, which makes it more promising for multiple sorption–regeneration cycles.

The difference in acid and alkali resistance is critically important for practical applications. This means that DBA–EChH–PEI will lose its capacity more slowly with multiple sorption-regeneration cycles, especially when using aggressive reagents. The higher capacity and thermal stability further strengthen its position. Due to its characteristics, DBA–EChH–PEI is particularly suitable for processes requiring multiple regeneration, operation under extreme pH conditions or at elevated temperatures. Its use can lead to lower operating costs and longer service life of the ion exchange column. Thus, DBA–EChH–PEI not only has slightly better performance, but also offers a qualitatively higher level of stability, which is a key factor for industrial applications.

**Figure 1:**
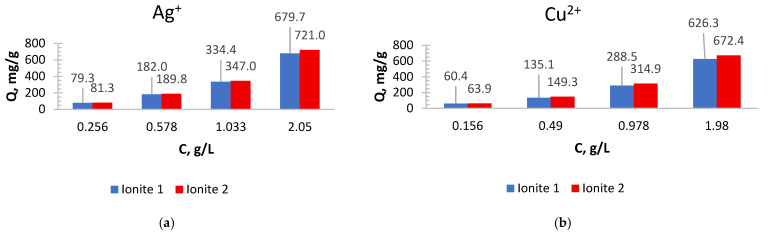
Sorption capacity depending on the concentration of solutions: (**a**) AgNO_3_; (**b**) Cu(NO_3_)_2_.

**Figure 2:**
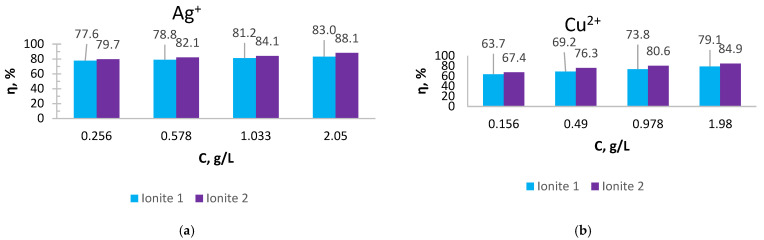
The degree of extraction of Ionites 1 and 2 in relation to ions: (**a**) AgNO_3_; (**b**) Cu(NO_3_)_2_.

**Figure 3:**
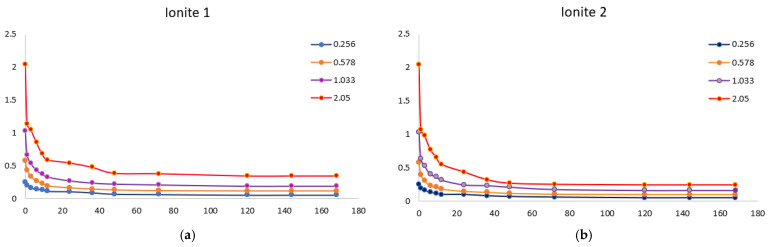
Silver ion concentration as a function of time by Ionites 1 (**a**) and 2 (**b**).

**Figure 4:**
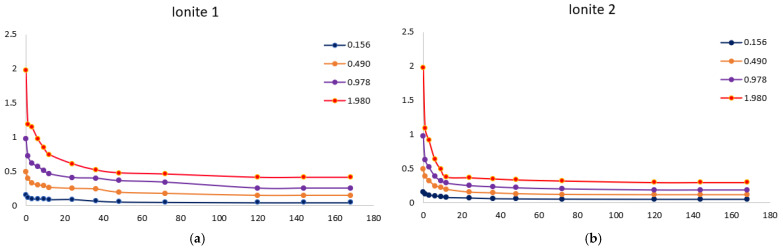
Copper ion concentration as a function of time by Ionites 1 (**a**) and 2 (**b**).

**Figure 5:**
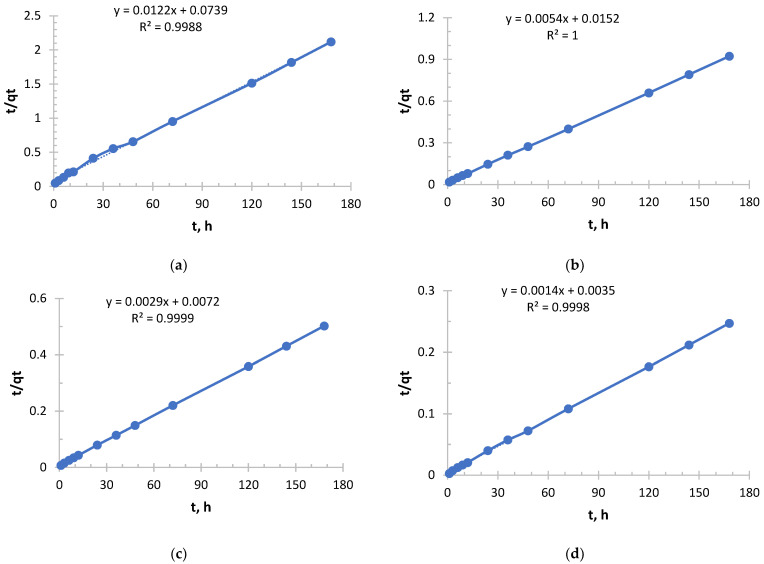
PSO kinetic model’s curve for Ionite 1 from silver nitrate solution. The initial concentrations are (**a**) c = 0.256 g/L; (**b**) c = 0.578 g/L; (**c**) c = 1.033 g/L; and (**d**) c = 2.050 g/L. Solid line is a real line for the kinetic model. Dash line is a trend line which is necessary to get curve equation for further calculations.

**Figure 6:**
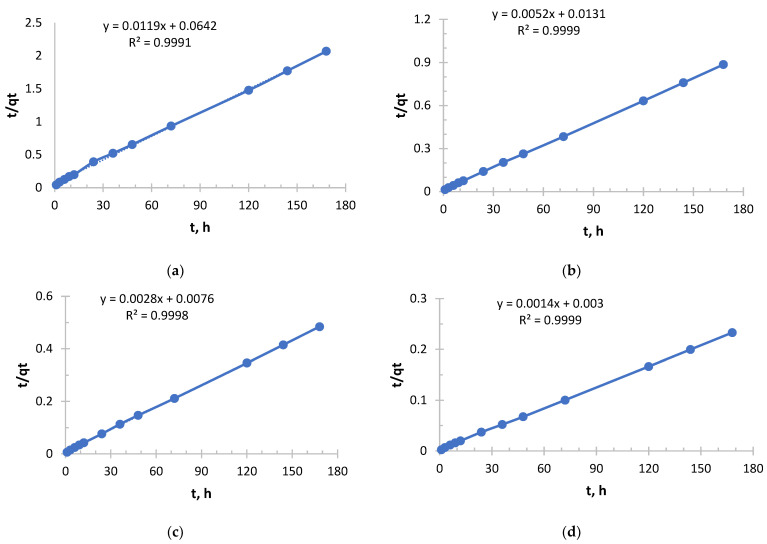
PSO kinetic model’s curve for Ionite 2 from silver nitrate solution. The initial concentrations are (**a**) c = 0.256 g/L; (**b**) c = 0.578 g/L; (**c**) c = 1.033 g/L; and (**d**) c = 2.050 g/L. Solid line is a real line for the kinetic model. Dash line is a trend line which is necessary to get curve equation for further calculations.

**Figure 7:**
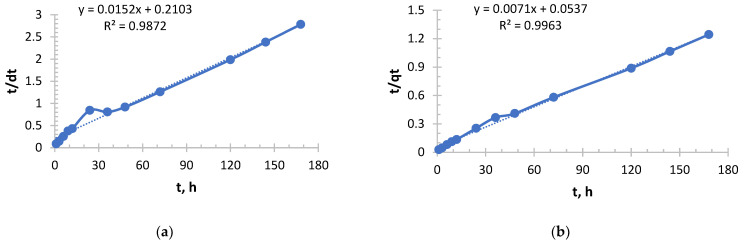

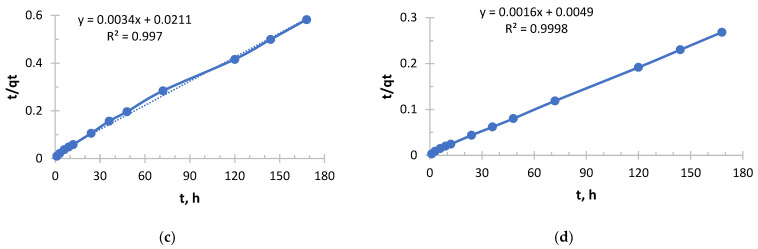
PSO kinetic model’s curve for Ionite 1 from copper nitrate solution. The initial concentrations are (**a**) c = 0.156 g/L; (**b**) c = 0.490 g/L; (**c**) c = 0.970 g/L; and (**d**) c = 1.980 g/L. Solid line is a real line for the kinetic model. Dash line is a trend line which is necessary to get curve equation for further calculations.

**Figure 8:**
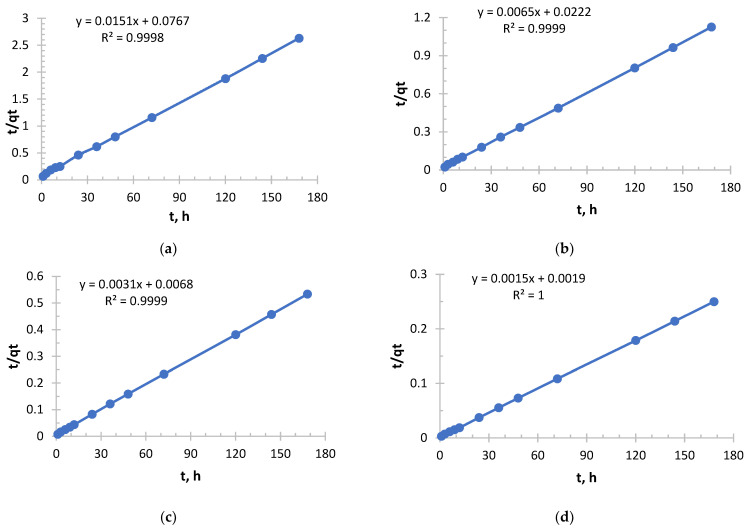
PSO kinetic model’s curve for Ionite 2 from copper nitrate solution. The initial concentrations are (**a**) c = 0.156 g/L; (**b**) c = 0.490 g/L; (**c**) c = 0.970 g/L; and (**d**) c = 1.980 g/L. Solid line is a real line for the kinetic model. Dash line is a trend line which is necessary to get curve equation for further calculations.

**Figure 9:**
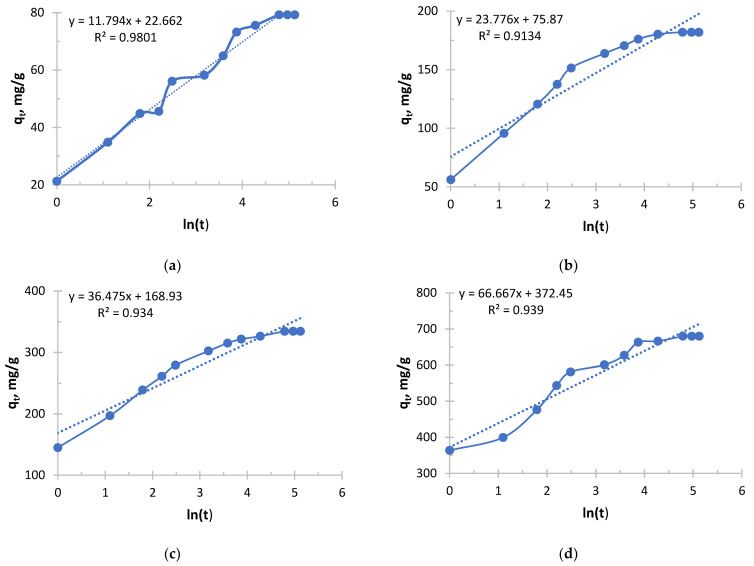
The Elovich kinetic model’s curve for Ionite 1 from silver nitrate solution. The initial concentrations are (**a**) c = 0.256 g/L; (**b**) c = 0.578 g/L; (**c**) c = 1.033 g/L; and (**d**) c = 2.050 g/L. Solid line is a real line for the kinetic model. Dash line is a trend line which is necessary to get curve equation for further calculations.

**Figure 10:**
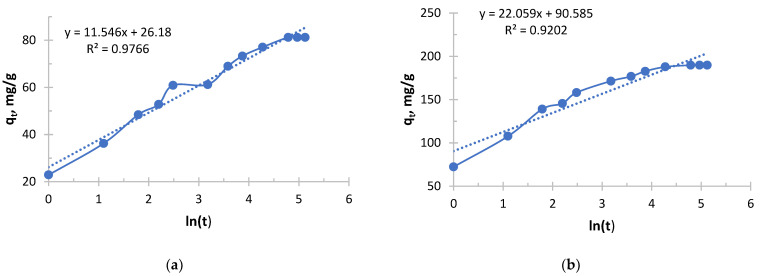

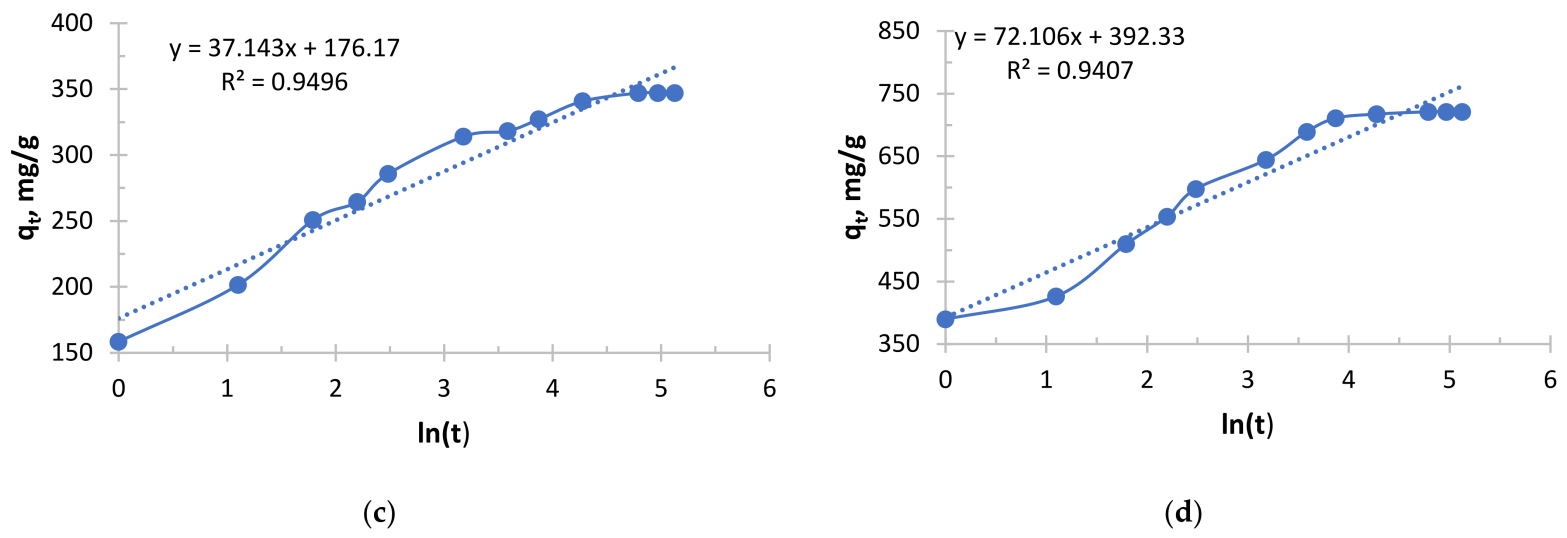
The Elovich kinetic model’s curve for Ionite 2 from silver nitrate solution. The initial concentrations are (**a**) c = 0.256 g/L; (**b**) c = 0.578 g/L; (**c**) c = 1.033 g/L; and (**d**) c = 2.050 g/L. Solid line is a real line for the kinetic model. Dash line is a trend line which is necessary to get curve equation for further calculations.

**Figure 11:**
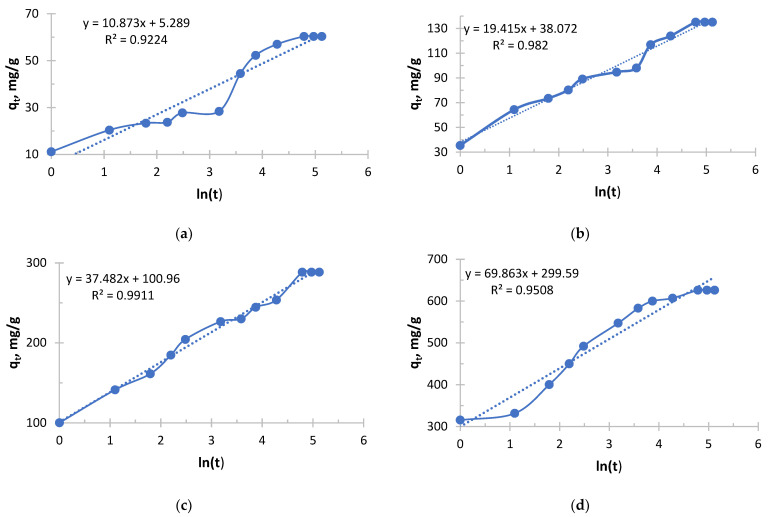
The Elovich kinetic model’s curve for Ionite 1 from copper nitrate solution. The initial concentrations are (**a**) c = 0.156 g/L; (**b**) c = 0.490 g/L; (**c**) c = 0.970 g/L; and (**d**) c = 1.980 g/L. Solid line is a real line for the kinetic model. Dash line is a trend line which is necessary to get curve equation for further calculations.

**Figure 12:**
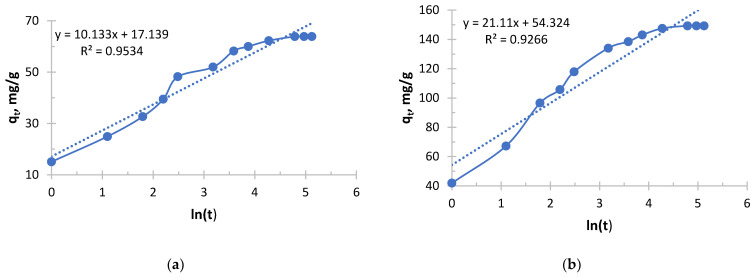

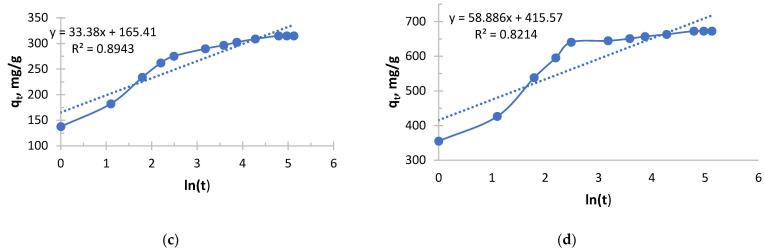
The Elovich kinetic model’s curve for Ionite 2 from copper nitrate solution. The initial concentrations are (**a**) c = 0.156 g/L; (**b**) c = 0.490 g/L; (**c**) c = 0.970 g/L; and (**d**) c = 1.980 g/L. Solid line is a real line for the kinetic model. Dash line is a trend line which is necessary to get curve equation for further calculations.

For the practical application of ionites, it is necessary to study the sorption of metal ions depending on the process conditions. In order to determine the optimal sorption parameters, the effect of the concentration (Figure 1 and Figure 2) and the duration of their contact (Figure 3, Figure 4, Figure 5, Figure 6, Figure 7, Figure 8, Figure 9, Figure 10, Figure 11 and Figure 12) with ionites (Ionite 1—Dowex HCR-S/S″, Ionite 2—DBA-EChH-PEI) on the extraction of silver (I) and copper (II) ions was studied.

As can be seen from Figure 1 and Figure 2, the SC of ionites increases with an increase in the content of silver and copper ions in solutions.

Ionite 2 has a higher sorption capacity in the extraction of Ag^+^ and Cu^2+^ ions compared to Ionite 1, the SC for silver ions is 679.7 and 721.0 mg/g and for copper ions 626.3 and 672.4 mg/g. The degree of extraction of silver ions reaches 83 and 88.1%, for copper ions—79.1 and 84.9%, respectively.

Figure 3 shows the isotherms of sorption of silver ions by Ionites 1 and 2. The equilibrium state between the ionites and the solution occurs after 120 h (5 days). The sorption process is characterized by the redistribution of metal ions from the bulk phase of the solution into the polymer matrix, which is reflected in the direct relationship between the concentration in the sorbent (capacity) and the inverse relationship with the equilibrium concentration in the solution.

Experiments show that the maximum sorption capacity of Ionite 1 shows a significant increase—from 79.3 mg/g to 679.7 mg/g—with an increase in the concentration of silver nitrate solution from 0.256 g/L to 2.050 g/L.

A clear positive correlation is observed for Ionite 2, where its maximum SC increases substantially with the AgNO_3_ concentration, reaching 721.0 mg/g at 2.050 g/L compared to 81.3 mg/g at the lowest concentration tested 0.256 g/L.

Figure 4 shows the isotherms of sorption of copper ions by Ionites 1 and 2. The equilibrium state between the ionites and the solution occurs after 120 h (5 days).

It was experimentally established that the maximum sorption capacity of Ionite 1 with respect to copper ions increased from 60.4 mg/g to 626.3 mg/g with an increase in the concentration of copper nitrate solution from 0.156 to 1.980 g/L.

A significant increase in the maximum sorption capacity of Ionite 2 was observed, from 63.9 mg/g to 672.4 mg/g, over the investigated range of copper nitrate concentrations (0.156–1.980 g/L).

As can be seen from Figure 3 and Figure 4, the sorption capacity of industrial ionite for silver ions is lower by 6% compared to synthesized, and for copper ions by 7%, which indicates a higher efficiency of synthesized ionite in the sorption of these ions and confirms the expediency of its use in purification systems where increased selectivity and capacity are required relative to silver and copper ions. The residual concentration curves demonstrate typical kinetic behavior for ion-exchange systems: a rapid initial sorption stage followed by a slower diffusion-limited phase, ultimately reaching equilibrium. The progressive decrease in concentration with time confirms the effective uptake of metal ions and the suitability of the sorbent for removing Cu^2+^ and Ag^+^ ions across a wide range of initial concentrations.

The extraction efficiency was determined for four initial metal-ion concentrations, covering dilute, medium, and elevated concentration ranges in order to simulate both wastewater-relevant and technologically relevant conditions. The synthesized DBA–EChH–PEI ionite and the commercial ion-exchange resin were examined under identical experimental settings to ensure comparability. The values presented in the table summarize the extraction degree (%) of each ionite at selected time points (1, 3, 6, 12, 15, 24, 48, 72, and 120 h). These data make it possible to visualize the differences in kinetic behavior between the two materials and to quantify the sorbent efficiency at each stage of the process. The full extraction data for both sorbents are presented in Table 2.

The time-resolved extraction profiles show that the synthesized DBA–EChH–PEI ionite exhibits superior kinetic behavior from the very beginning of the sorption process. Within the first 1–3 h, it extracts 4–8% more Ag^+^ and 6–10% more Cu^2+^ compared with the industrial ionite. After 6–12 h, the difference becomes more pronounced, and after 15 h the synthesized ionite reaches up to 60–72% extraction depending on concentration, whereas the industrial material remains 10–15% lower. At longer intervals (24–72 h), Ionite 2 maintains its advantage, demonstrating slower saturation of active sites and more stable extraction rates. At 120 h the synthesized ionite achieves 79–88% extraction for Ag^+^ and 67–85% for Cu^2+^ across the studied concentration range, consistently outperforming the commercial ionite. These results confirm the enhanced kinetics and higher overall efficiency of the DBA–EChH–PEI ionite.

The analytical uncertainty of the concentration determination method (3–5%) contributes to the dispersion observed in the isotherm data. For the pair of ionites where the difference in sorption capacity is approximately 7–8%, this methodological error renders the distinction marginal. Within the accuracy of the applied experimental procedure, the sorption properties of the two ionites can therefore be considered statistically indistinguishable.

Sorption of heavy metal ions by synthesized anionites can be explained by the formation of coordination bonds by the donor-acceptor mechanism between the electron-donor groups of the ionite (-NH2, =NH, ≡N) and the vacant orbital of the transition metal ion [33]:

≡ N: + M^n+^ → N≡ : M^n+^

The process of industrial ionite extraction proceeds according to the following scheme, where metal ions are exchanged on the cationite in the H^+^ form:

(R-SO_3_^−^H^+^) + M ^n+^ ↔ (R-SO_3_^−^)_n_M^n+^ + 2H^+^

In order to evaluate the sorbents (Ionite 1 and Ionite 2) Pseudo-Second order (PSO) kinetic model and Elovich kinetic model were used.

Pseudo-second order and Elovich models were used to analyze the sorption kinetics [17]. The pseudo-second-order model was used to estimate the maximum sorption capacity and the rate constant of the process, as well as to test the hypothesis of a limiting stage associated with chemical interaction. The Elovich model was used to analyze the kinetics throughout sorption in order to confirm the significant contribution of chemosorption and to evaluate the parameters characterizing the initial rate of the process and the activation energy.

The pseudo-Second order kinetic model is shown in Figure 5, Figure 6, Figure 7 and Figure 8.

From Figure 5 and Figure 6 and Table 3 and Table 4, it follows that with increasing concentration, a rapid increase in the initial sorption rate is observed. With an increase in concentration from 0.256 to 2.050 g/L, the rate increased by 20 times, which is a direct consequence of an increase in the driving force of the process—the concentration gradient. The higher the concentration, the more Ag^+^ ions “attack” the active centers of the sorbent per unit time.

The value of the rate constant steadily decreases with increasing initial concentration due to the fact that at low concentrations, ions primarily occupy the most accessible and energetically advantageous active centers. In concentrated solutions, these “best” centers fill up very quickly, and further sorption occurs at less accessible centers (requiring, for example, diffusion deep into the pores), which is a slower process and reduces the average rate constant.

Depending on the concentration, sorption took place in different ways, demonstrating a strong dependence on concentration. In a solution with a lower concentration (0.256 g/L), the process was slowest, but with maximum “specific” efficiency. This is a regime that is close to ideal for the active centers of the sorbent. In a solution with a higher concentration (2.050 g/L), sorption started extremely intensively, but its overall efficiency was the lowest. This is the “saturation” mode, when the kinetics begins to be limited not only by chemical interaction, but also by the availability of active centers.

For both sorbents, the choice of optimal conditions depends on the purpose:

- To quickly extract large amounts of silver, it is more profitable to use more concentrated solutions, since the initial velocity is at its maximum here.
- In order to achieve maximum sorbent efficiency and, possibly, more complete extraction from dilute solutions, conditions with a lower concentration, where the rate constant k_2_ is higher, may be preferable.

The sorption of silver ions by both ion exchange resins was carried out in full accordance with theoretical expectations. An increase in concentration accelerates the process at the start, but reduces the overall efficiency of the sorption mechanism, described by the constant k_2_.

As can be seen from Figure 7 and Table 5, it follows that an exponential increase in the initial sorption rate is observed with increasing concentration. With an increase in concentration from 0.156 to 1.980 g/L, the rate increased by more than 40 times. This is a consequence of an increase in the driving force of the process, the concentration gradient: the higher the concentration of copper ions (Cu^2+^), the more they diffuse to the surface of the sorbent and interact with active centers per unit time.

The value of the velocity constant decreases with increasing initial concentration, which is consistent with the general theory. However, it is important to note here that the most significant drop in k_2_ occurs between solutions with concentrations of 0.490 and 0.978 g/L, while the rate constant decreases slightly between solutions with concentrations of 0.978 and 1.980. This may indicate that at concentrations above 1.0 g/L, the mechanism or the limiting stage of the process stabilizes.

The sorption of copper, like silver with Ionite 1, follows general patterns: with increasing concentration, the initial velocity increases; the rate constant k_2_ decreases monotonously.

For all the corresponding concentrations, the initial sorption rate of silver ions is significantly higher than that of copper ions. For example, for a concentration of about 2.0 g/L: V_0_(Ag) = 333.33 mg/g·h, and v_0_(Cu) = 204.08 mg/g·h. This suggests that the sorbent used has a higher affinity for silver ions or that the kinetics of their interaction is faster.

The k_2_ values for copper are about 2–3 times lower than for silver at the same concentrations. This confirms the conclusion that the silver sorption process on this sorbent proceeds more efficiently.

The successful description of the kinetics by the pseudo-second-order model indicates that the limiting stage is chemisorption, probably involving ion exchange or the formation of coordination bonds between copper ions and sorbent functional groups.

Copper sorption was predictable, demonstrating a strong dependence on concentration. Compared with silver, copper sorption on this sorbent is characterized by a lower rate and overall efficiency. This is an important practical conclusion that must be taken into account when designing cleaning processes for solutions containing metal mixtures.

From Figure 8 and Table 6, it can be seen that an extremely sharp increase in the initial sorption rate is observed with increasing concentration. With an increase in concentration from 0.156 to 1.980 g/L, the rate increased 40-fold. This indicates a very strong dependence of the rate of the process on the concentration, that is, a high “order” of reaction for the substance in solution. The driving force of the process (concentration difference) is a key factor.

The value of the rate constant decreases with increasing initial concentration, which is expected. However, it is important to note the slowing decline of k_2_. The greatest jump in efficiency reduction occurs when switching from the lowest concentration (0.156 g/L) to a higher one (0.490 g/L). With a further increase in concentration, the drop in k_2_ becomes less pronounced. This may mean that at concentrations above 0.5 g/L, the system enters a stable mode where the sorption mechanism does not change so dramatically.

In this experiment, copper sorption is characterized by very high rates, especially in concentrated solutions. The initial velocity in a solution with a concentration of 0.1980 mg/L (526.32 mg/g·h) is more than 2.5 times higher than in a similar solution from a previous experiment using Ionite 1 (204.08 mg/g·h). This suggests that the kinetics of copper sorption has improved significantly under these conditions.

The values of the velocity constant in this experiment using Ionite 2 are also significantly higher (2–3 times) than in the case of Ionite 1. This indicates that in this case, the sorption process was generally more efficient at all concentrations.

In a solution with a concentration of 0.156 g/L, the process proceeded with maximum “specific” efficiency (highest k_2_), but at a moderate speed.

In a solution with a concentration of 1.980 g/L, sorption started extremely intensively (a record V_0_), demonstrating the sorbent’s ability to absorb large amounts of copper from concentrated solutions very quickly, although the overall efficiency (k_2_) was lower.

The data from this experiment is much more favorable for practical applications. They show that this particular sorbent is very well adapted under these conditions for the rapid extraction of copper from medium and highly concentrated solutions.

The copper sorption in this experiment was much more efficient and faster than in the previous data. A classic but very pronounced pattern is observed: a sharp increase in velocity and a gradual decrease in the velocity constant with increasing concentration. The sorbent demonstrated high kinetic activity with respect to copper ions. The largest amount of copper in absolute terms was extracted from the most concentrated solution with a concentration of 1.980 g/L. At the same time, the extraction efficiency remained consistently high for both dilute and concentrated solutions, which makes this sorbent promising for use in a wide range of initial copper concentrations, from wastewater treatment to extraction of valuable metal from technological solutions.

For both metals, especially in concentrated solutions, the initial sorption rate (v_0_) for DBA-EHG-PEI ionite is significantly higher than the rate of an industrial sorbent: for copper–2.6 times, for silver–1.2 times, that is, the synthesized ionite “captures” metal ions from the solution faster at the initial stage, which is It is a good indicator for technological processes where it is important to quickly reduce the concentration of metal.

In experiments with copper and silver, the k_2_ value for DBA-EChH-PEI is higher than that for Dowex, which indicates that the process of chemical interaction (chemisorption) with the active centers of DBA-EChH-PEI proceeds more efficiently during the entire contact time. This means not only a fast start, but also more optimal kinetics at all stages until equilibrium is reached.

The Elovich kinetic model is shown in Figure 9, Figure 10, Figure 11 and Figure 12.

Silver sorption took place in radically different ways depending on the concentration, and the Elovich equation clearly demonstrates this from Figure 9 and Figure 10 and Table 7 and Table 8.

The process in more concentrated solutions started at a rate an order of magnitude higher than in less concentrated ones. High values of the α parameter show that the driving force of the process (concentration) is the decisive factor at the start. Even an increase in the concentration from 0.256 to 0.578 g/L leads to a tremendous increase in α.

Sorption in concentrated solutions was not only faster at the start, but also significantly more stable. A 6.2-fold decrease in the β parameter means that the sorption rate decreased much more slowly as the active centers were filled. This indicates that the high concentration of silver ions effectively compensates for the increasing difficulty of accessing the remaining active centers.

In the solution with the lowest concentration of 0.256 g/L, the process started at a relatively low speed and quickly exhausted easily accessible centers, slowing down sharply.

In the solution with the highest concentration of 2.050 g/L, sorption began with maximum intensity and maintained a high rate for a much longer time, allowing the sorbent to realize its full kinetic potential and, probably, a large capacity.

The analysis of the Elovich equation clearly shows that the use of this sorbent for the extraction of silver from concentrated solutions is highly effective. Concentrated solutions provide both maximum initial velocity and longer and more stable sorption, making the process more intensive and probably more productive.

It follows from Figure 11 and Figure 12 and Table 9 and Table 10 that copper sorption with Ionite 2 was characterized by a strong dependence of kinetic parameters on the initial concentration. The key parameter, α, increased exponentially with its increase, reflecting the difference in the initial rate of the process by more than 170 orders of magnitude between the limiting concentrations. In parallel, a steady decrease in the β parameter was observed, which indicates a slower depletion of sorption capacity in concentrated solutions. In this case, the high concentration of copper ions compensates for the increasing difficulty of accessing the remaining active centers of the polymer. As a result, in a solution with a maximum concentration (1.980 g/L), the process was characterized by a high initial speed and stability, whereas in a dilute solution (0.156 g/L), sorption slowed down rapidly after the exhaustion of easily accessible centers. Therefore, high initial concentrations of copper are necessary for the effective manifestation of the kinetic properties of this sorbent.

Analysis of the Elovich model shows that the high concentration of ions in the solution better “compensates” for the increasing difficulty of accessing the remaining active centers. The sorbent retains its high activity for longer, which makes it possible to use its full capacity more efficiently.

Although DBA-EChH-PEI starts faster, it takes the same amount of time for it to fully saturate as its industrial counterpart. This may be due to the stage of slow intradiffusion kinetics in the late stages of the process, which limits both materials.

## 4. Conclusions

A multifunctional polymer ionite based on DBA–EChH–PEI was successfully synthesized. The obtained material contains a high density of active functional groups and demonstrates chemical stability in acidic and alkaline media, confirming its suitability for repeated sorption–desorption cycles.

The sorption behavior of the synthesized ionite was evaluated in comparison with an industrial ionite. The DBA–EChH–PEI material exhibited higher equilibrium sorption capacities, faster initial sorption rates, and more stable kinetic performance. These results indicate improved accessibility and reactivity of the active centers formed within the polymer network.

Ionite 2 demonstrated increased sorption capacities for Cu^2+^ (672.4 mg/g) and Ag^+^ (721.0 mg/g) compared to the commercial ionite (626.3 mg/g and 679.7 mg/g, respectively). The material also showed a higher initial sorption rate (V_0_) and slower decline in sorption performance over time, indicating more efficient utilization of available active sites. The obtained data support the suitability of this ionite for processing both concentrated technological solutions and diluted wastewater streams.

Kinetic analysis based on the pseudo-second-order and Elovich models confirmed the high reactivity of the synthesized material.

The pseudo-second-order model indicated that increasing the initial metal concentration enhances the overall sorption rate, while the rate constant decreases gradually, reflecting changes in mass-transfer conditions.

The Elovich model provided insight into microkinetics, showing reduced β values at higher concentrations, which suggests prolonged activity of the ionite and effective interaction with metal ions even at later stages of sorption.

Both models consistently demonstrated the superior kinetic characteristics of the DBA–EChH–PEI ionite compared with the industrial ionite.

Overall, the synthesized DBA–EChH–PEI ionite shows improved sorption and kinetic characteristics toward Cu^2+^ and Ag^+^ ions. The combination of high sorption capacity, enhanced kinetic parameters, and chemical stability suggests that the material is a promising candidate for applications involving the recovery of valuable metals from technological solutions and industrial wastewater.

## Figures and Tables

**Table 1:** Physico-chemical characteristics of synthesized ionite.

Anion Exchanger	SEC_HCl_, mg-equiv/g	*V*_sp_, mL/g	Chemical Stability in Solutions, %			Thermal Stability in Water, %
			5 N H_2_SO_4_	5 N NaOH	10% H_2_O_2_	
DBA–EChH–PEI	9.20	4.9	97.7	98.4	71.4	94.4
Dowex HCR-S/S”	8.03	4.5	85.5	86.1	70.2	89.0

**Table 2:** Time-dependent extraction degree (%) of Cu^2+^ and Ag^+^ ions by the commercial ionite Dowex (Ionite 1) and the synthesized DBA–EChH–PEI ionite (Ionite 2) at different initial metal concentrations.

**Ag^+^**											
**C, g/L**	**T, h**	**1**	**3**	**6**	**9**	**12**	**24**	**36**	**48**	**72**	**120**
0.256	Ionite 1	20.83	34.11	43.88	44.66	54.94	56.99	63.61	71.69	74.02	77.6
	Ionite 2	22.42	35.55	47.51	51.77	59.73	60.03	67.65	71.91	75.62	79.7
0.578	Ionite 1	24.34	41.46	52.25	59.57	65.63	70.99	73.88	76.24	78.09	78.84
	Ionite 2	31.3	46.6	60.15	62.98	68.4	74.16	76.47	79.07	81.26	82.09
0.978	Ionite 1	35.2	47.82	58.02	63.44	67.8	73.38	76.51	78.06	79.25	81.19
	Ionite 2	38.38	48.82	60.76	64.06	69.25	76.09	77.09	79.24	82.58	84.08
2.05	Ionite 1	44.41	48.8	58.18	66.38	70.96	73.33	76.52	80.96	81.33	82.99
	Ionite 2	47.63	52.11	62.31	67.67	73.11	78.76	84.26	86.9	87.69	88.14
**Cu^2+^**											
**C, g/L**	**T, h**	**1**	**3**	**6**	**9**	**12**	**24**	**36**	**48**	**72**	**120**
0.156	Ionite 1	11.79	21.53	24.61	25.04	29.25	29.96	46.98	55.13	60.14	63.68
	Ionite 2	15.92	26.3	34.46	41.63	50.88	54.85	61.47	63.32	65.72	67.42
0.490	Ionite 1	18.19	32.99	37.63	41.11	45.66	48.51	50.23	59.89	63.48	69.18
	Ionite 2	21.42	34.37	49.36	54.06	60.26	68.5	70.76	73.09	75.39	76.27
1.03	Ionite 1	25.6	36.16	41.27	47.21	52.25	57.94	58.82	62.52	64.86	73.77
	Ionite 2	35.19	46.59	59.79	66.95	70.37	74.11	75.83	77.33	78.97	80.57
1.98	Ionite 1	39.83	41.89	50.57	56.87	62.15	69.11	73.62	75.77	76.68	79.11
	Ionite 2	44.83	53.81	67.91	75.18	80.84	81.36	82.16	82.92	83.72	84.9

**Table 3:** Calculated data on the sorption of silver ions by Ionite 1 using the PSO curve.

Concentration, C_0_ (g/L)	0.256	0.578	1.033	2.050
Sorbent mass, g	0.2506	0.2503	0.2508	0.2503
Volume of solution, L	0.1	0.1	0.1	0.1
k_2_, g/mg·h	0.002014	0.001918	0.001168	0.000560
v_0_, mg/g·h	13.53	65.79	138.89	285.71

**Table 4:** Calculated data on the sorption of silver ions by Ionite 2 using the PSO curve.

Concentration, C_0_ (g/L)	0.256	0.578	1.033	2.050
Sorbent mass, g	0.2511	0.2500	0.2503	0.2506
Volume of solution, L	0.1	0.1	0.1	0.1
k_2_, g/mg·h	0.002206	0.002064	0.001032	0.000653
v_0_, mg/g·h	15.58	76.34	131.58	333.33

**Table 5:** Calculated data on the sorption of copper ions by Ionite 1 using the PSO curve.

Concentration, C_0_ (g/L)	0.156	0.490	0.978	1.980
Sorbent mass, g	0.2500	0.2509	0.2501	0.2501
Volume of solution, L	0.1	0.1	0.1	0.1
k_2_, g/mg·h	0.001099	0.000939	0.000548	0.000522
v_0_, mg/g·h	4.76	18.62	47.39	204.08

**Table 6:** Calculated data on the sorption of copper ions by Ionite 2 using the PSO curve.

Concentration, C_0_ (g/L)	0.156	0.490	0.978	1.980
Sorbent mass, g	0.2502	0.2504	0.2502	0.2500
Volume of solution, L	0.1	0.1	0.1	0.1
k_2_, g/mg·h	0.002973	0.001903	0.001413	0.001184
v_0_, mg/g·h	13.04	45.05	147.06	526.32

**Table 7:** Calculated data on the sorption of silver ions by Ionite 1 using the Elovich kinetic model’s curve.

Concentration C_0_, g/L	0.256	0.578	1.033	2.050
Slope B (1/β)	11.794	23.776	36.475	66.667
Intercept A	22.662	75.87	168.93	372.45
α, mg/g·h	7.05 × 10^9^	9.35 × 10^32^	2.58 × 10^73^	7.16 × 10^161^
Surface coverage β, g/mg	0.0848	0.0421	0.0274	0.0150

**Table 8:** Calculated data on the sorption of silver ions by Ionite 2 using the Elovich kinetic model’s curve.

Concentration C_0,_ g/L	0.256	0.578	1.033	2.050
Slope B (1/β)	11.546	22.059	37.143	72.106
Intercept A	26.18	90.585	176.17	392.33
α, mg/g·h	2.38 × 10^11^	2.32 × 10^39^	3.61 × 10^76^	3.12 × 10^170^
Surface coverage β, g/mg	0.0866	0.0453	0.0269	0.0139

**Table 9:** Calculated data on the sorption of copper ions by Ionite 1 using the Elovich kinetic model’s curve.

Concentration C_0_, g/L	0.156	0.490	0.978	1.980
Slope B (1/β)	10.873	19.415	37.482	69.863
Intercept A	5.289	38.072	100.96	299.59
α, mg/g·h	198.81	3.51 × 10^16^	7.48 × 10^43^	1.56 × 10^130^
Surface coverage β, g/mg	0.0920	0.0515	0.0267	0.0143

**Table 10:** Calculated data on the sorption of copper ions by Ionite 2 using the Elovich kinetic model’s curve.

Concentration C_0_, g/L	0.156	0.490	0.978	1.980
Slope B (1/β)	10.133	21.110	33.380	58.886
Intercept A	17.139	54.324	165.41	415.57
α, mg/g·h	2.81 × 10^7^	4.05 × 10^23^	7.62 × 10^71^	3.92 × 10^180^
surface coverage β, g/mg	0.0920	0.0515	0.0267	0.0143

## Data Availability

The original contributions presented in this study are included in the article. Further inquiries can be directed to the corresponding author.

## Abbreviations

The following abbreviations are used in this manuscript:

DBA	Multidisciplinary Digital Publishing Institute
EChH	Directory of open access journals
PEI	Three-letter acronym
